# [1,2-Bis­(diisopropyl­phosphan­yl)ethane-κ^2^ *P*,*P*′]dichloridonickel(II)–9*H*-carbazole (1/2)

**DOI:** 10.1107/S1600536811010555

**Published:** 2011-03-26

**Authors:** Farah Cañavera-Buelvas, Marcos Flores-Alamo, Juventino J. García

**Affiliations:** a Facultad de Química, Universidad Nacional Autónoma de México, México DF 04510, Mexico

## Abstract

In the title compound, [NiCl_2_(C_14_H_32_P_2_)]·2C_12_H_9_N, the neutral [Ni(dppe)Cl_2_] complex [dppe is 1,2-bis­(diisopropyl­phosphan­yl)ethane] consists of a tetracoordinated Ni^2+^ cation and has a crystallographic twofold axis passing through the metal atom and the mid-point of the CH_2_—CH_2_ bond of the dppe ligand. The metal atom shows slight tetra­hedral distortion from an ideal square-planar coordination geometry, as reflected in the dihedral angle between NiCl_2_ and NiP_2_ planes of 15.32 (2)°. The 9*H*-carbazole ring system is essentially planar (r.m.s. deviation = 0.022 Å). In the crystal packing, there are two symmetry-related 9*H*-carbazole mol­ecules between two adjacent Ni^II^ complexes, with an angle between the carbazole mean planes of *ca* 77°.

## Related literature

For the use of nickel complexes of the type [Ni(dppe)Cl_2_] as starting materials and precursors in metal-mediated and catalytic systems, respectively, see: Vicic & Jones (1997 ▶); Arévalo & García (2010 ▶). For details of tetra­hedral distortion and motifs, see: Angulo *et al.* (2003 ▶); Dahlenburg & Kurth (2001 ▶); Etter *et al.* (1990 ▶).
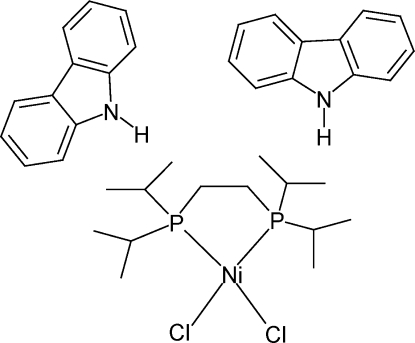


## Experimental

### 

#### Crystal data

[NiCl_2_(C_14_H_32_P_2_)]·2C_12_H_9_N
*M* *_r_* = 726.35
Monoclinic, 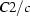

*a* = 22.5830 (5) Å
*b* = 8.4374 (2) Å
*c* = 18.9630 (5) Å
β = 101.544 (2)°
*V* = 3540.15 (15) Å^3^
*Z* = 4
Mo *K*α radiation
μ = 0.82 mm^−1^
*T* = 122 K
0.42 × 0.16 × 0.02 mm

#### Data collection

Oxford Diffraction Xcalibur Atlas Gemini diffractometer
Absorption correction: analytical [*CrysAlis PRO* (Oxford Diffraction, 2009 ▶); based on expressions derived by Clark & Reid (1995 ▶)] *T*_min_ = 0.851, *T*_max_ = 0.987
12792 measured reflections
3484 independent reflections
2908 reflections with *I* > 2σ(*I*)
*R*_int_ = 0.027

#### Refinement

*R*[*F* ^2^ > 2σ(*F* ^2^)] = 0.026
*wR*(*F* ^2^) = 0.065
*S* = 1.05
3484 reflections
211 parameters
H atoms treated by a mixture of independent and constrained refinement
Δρ_max_ = 0.62 e Å^−3^
Δρ_min_ = −0.26 e Å^−3^

### 

Data collection: *CrysAlis CCD* (Oxford Diffraction, 2009 ▶); cell refinement: *CrysAlis RED* (Oxford Diffraction, 2009 ▶); data reduction: *CrysAlis RED*; program(s) used to solve structure: *SHELXS97* (Sheldrick, 2008 ▶); program(s) used to refine structure: *SHELXL97* (Sheldrick, 2008 ▶); molecular graphics: *ORTEP-3 for Windows* (Farrugia, 1997 ▶); software used to prepare material for publication: *WinGX* (Farrugia, 1999 ▶).

## Supplementary Material

Crystal structure: contains datablocks I, global. DOI: 10.1107/S1600536811010555/bh2344sup1.cif

Structure factors: contains datablocks I. DOI: 10.1107/S1600536811010555/bh2344Isup2.hkl

Additional supplementary materials:  crystallographic information; 3D view; checkCIF report

## Figures and Tables

**Table:** 

Ni1—Cl1	2.2221 (4)
Ni1—P1	2.1581 (5)

**Table:** 

P1^i^—Ni1—P1	88.61 (3)
P1^i^—Ni1—Cl1^i^	168.757 (16)
P1^i^—Ni1—Cl1	89.797 (16)
Cl1^i^—Ni1—Cl1	93.87 (2)

Symmetry code: (i) 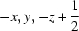.

**Fig. 1.**
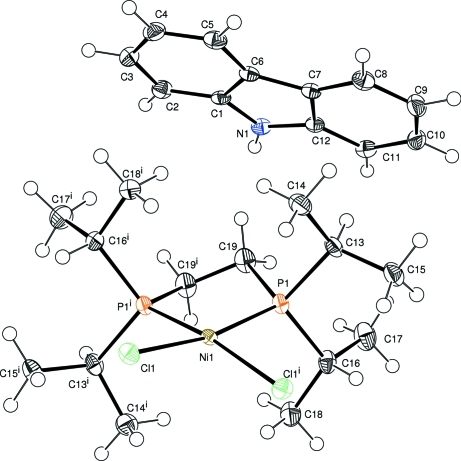
The molecular structure for the title compound. Displacement ellipsoids are draw at the 50% probability level and H atoms are shown as circles of arbitrary size. Symmetry code for i labeled atoms: -x, y, -z + 1/2.

**Fig. 2.**
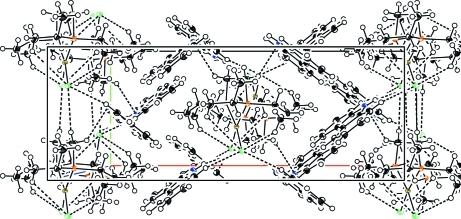
Intermolecular van der Waals contacts (dashed lines) in the crystal structure, forming infinite ribbons including R12(5) motifs.

## supplementary crystallographic information

### Comment

The nickel complexes of the type [Ni(dppe)Cl_2_] are useful starting materials for the preparation of catalysts and catalytic precursors, for an interesting series of active catalyst in a wide variety of stoichiometric (Vicic & Jones, 1997) and catalytic systems (Arévalo & García, 2010). The synthesis of the current complex [Ni (dppe)Cl_2_](carbazole)_2_ (see Scheme) can be envisaged as the preparation of a model compound relevant to hydrodenitrogenation and N—H activation process.

In the asymmetric unit, the tetracoordinated [Ni(dppe)Cl_2_] complex has a 2-fold axis passing through the metal and the centre of the methylene—methylene bond (Fig. 1). The metal center shows slight tetrahedral distortion from ideal square-planar coordination geometry, with the angle between the normals to the planes defined by the two *cis*-Cl–Ni–Cl and *cis*-P–Ni–P fragments [15.32 (2)°] being larger than the limiting value of 0° for square-planar coordination. Additionally, the metal ion is situated 0.1144 (1) Å above the Cl1/P1/Cl1*^i^*/P1*^i^* plane [symmetry code: (*i*) -*x*, *y*, 1/2 - *z*]. These deviations from planarity, which can safely be attributed to some steric congestion by intermolecular contacts between the metallic complex and the 9*H*-carbazole molecules (Fig. 2), are somewhat larger than the distortion from ideal square-planar coordination geometry observed for related [Ni(*dcpe*)Cl_2_] (Angulo *et al.*, 2003) and [(1*S*,2*S*)-C_5_H_8_{P(C_6_H_11_)_2_}_2_NiCl_2_] (Dahlenburg & Kurth, 2001) complexes, where the NiCl_2_/NiP_2_ dihedral angles are 3.96 and 5.37°, respectively. The Ni–P bond lengths in the title compound are equal, by symmetry. Probably as a consequence of the steric bulk of the 9*H*-carbazole molecules, the Ni–Cl distance, 2.2221 (4) Å, tends to be slightly longer than those in the analogues nickel complexes.

In the crystal packing, there are two 9*H*-carbazole molecules between two adjacent nickel complexes, with an angle between the carbazole mean planes of *ca*. 77°. There are two types of intermolecular contacts: one of the van der Waals type N—H···Cl [2.65 (2) Å] is formed from N1 donor atom of the 9*H*-carbazole to Cl1 chloride atom acceptor of the metallic complex; and other of the type C—H···N [2.645 (1) Å] involving C19 in the dppe ligand and N1 in 9*H*-carbazole. These van der Waals interactions lead to infinite ribbons based on *R^1^_2_*(5) motifs (Etter *et al.*, 1990), as illustrated in Fig. 2.

### Experimental

A THF solution of [Ni(dppe)H]_2_ (Vicic & Jones, 1997) (0.100 g, 0.15 mmol) was added with 9*H*-carbazole (0. 261 g, 1.56 mmol) and heated to 80 °C for 10 h. After this time, the solution changed from wine red color to brown. The solvent was eliminated under reduced pressure and the resulting solid dissolved in dichloromethane (DCM). Slow evaporation at room temperature of DCM afforded crystals suitable for X-ray diffraction. NMR: ^31^P{^1^H} (acetone-*d*_6_,121.32 MHz, 25 °C): d 57.2. NMR ^1^H (acetone-*d*_6_, 300 MHz, 25 °C): d 8.13 (d, *J*_H—H_=7.8, 1H), d 7.54 (d, *J*_H—H_=7.8, 1H), d 7.39 (dd, *J*_H—H_=7.8, *J*_H—H_=7.2, 1H), d 7.18 (dd, *J*_H—H_=7.2, *J*_H—H_=7.8, 1H), d 2.5 (m, CH, 2H), d 1.7 (m, CH_2_, 2H), d 1.35 (m, CH_3_, 12H). Elemental analysis (calc.): C 62.9 (62.83), H 6.90 (6.93), N 3.82% (3.85%).

### Refinement

H atom bonded to N atom was located in a difference and was refined with free coordinates and *U*_iso_(H) = 1.2*U*_eq_(N). H atoms attached to C atoms were placed in geometrically idealized positions, and refined as riding on their parent atoms, with C—H distances fixed to 0.95 (aromatic CH), 0.98 (methyl CH_3_), 0.99 (methylene CH_2_) and 1.00 Å (methine CH), and with *U*_iso_ = 1.2–1.5 *U*_eq_(C).

### Crystal data

**Table:**

[NiCl_2_(C_14_H_32_P_2_)]·2C_12_H_9_N	*F*(000) = 1536
*M**_r_* = 726.35	*D*_x_ = 1.363 Mg m^−^^3^
Monoclinic, *C*2/*c*	Mo *K*α radiation, λ = 0.71073 Å
Hall symbol: -C 2yc	Cell parameters from 7654 reflections
*a* = 22.5830 (5) Å	θ = 3.3–26.0°
*b* = 8.4374 (2) Å	µ = 0.82 mm^−^^1^
*c* = 18.9630 (5) Å	*T* = 122 K
β = 101.544 (2)°	Prism, orange
*V* = 3540.15 (15) Å^3^	0.42 × 0.16 × 0.02 mm
*Z* = 4	

### Data collection

**Table:**

Oxford Diffraction Xcalibur Atlas Gemini diffractometer	3484 independent reflections
Radiation source: fine-focus sealed tube	2908 reflections with *I* > 2σ(*I*)
graphite	*R*_int_ = 0.027
Detector resolution: 10.4685 pixels mm^-1^	θ_max_ = 26.1°, θ_min_ = 3.5°
ω scans	*h* = −27→27
Absorption correction: analytical [*CrysAlis PRO* (Oxford Diffraction, 2009); based on expressions derived by Clark & Reid (1995)]	*k* = −10→10
*T*_min_ = 0.851, *T*_max_ = 0.987	*l* = −18→23
12792 measured reflections	

### Refinement

**Table:**

Refinement on *F*^2^	Primary atom site location: structure-invariant direct methods
Least-squares matrix: full	Secondary atom site location: difference Fourier map
*R*[*F*^2^ > 2σ(*F*^2^)] = 0.026	Hydrogen site location: inferred from neighbouring sites
*wR*(*F*^2^) = 0.065	H atoms treated by a mixture of independent and constrained refinement
*S* = 1.05	*w* = 1/[σ^2^(*F*_o_^2^) + (0.0343*P*)^2^ + 0.7966*P*] where *P* = (*F*_o_^2^ + 2*F*_c_^2^)/3
3484 reflections	(Δ/σ)_max_ = 0.001
211 parameters	Δρ_max_ = 0.62 e Å^−^^3^
0 restraints	Δρ_min_ = −0.25 e Å^−^^3^
0 constraints	

### Fractional atomic coordinates and isotropic or equivalent isotropic displacement parameters (Å^2^)

**Table:**

	*x*	*y*	*z*	*U*_iso_*/*U*_eq_	
C1	0.13521 (7)	0.51654 (19)	0.17978 (9)	0.0162 (4)	
C2	0.10421 (8)	0.4878 (2)	0.10949 (9)	0.0208 (4)	
H2	0.0704	0.4185	0.0998	0.025*	
C3	0.12477 (8)	0.5644 (2)	0.05455 (10)	0.0237 (4)	
H3	0.1046	0.5469	0.0062	0.028*	
C4	0.17440 (8)	0.6668 (2)	0.06818 (10)	0.0231 (4)	
H4	0.1873	0.7177	0.0291	0.028*	
C5	0.20488 (8)	0.6947 (2)	0.13744 (10)	0.0207 (4)	
H5	0.2385	0.7648	0.1464	0.025*	
C6	0.18570 (7)	0.61860 (19)	0.19446 (9)	0.0163 (4)	
C7	0.20640 (7)	0.61872 (19)	0.27183 (9)	0.0164 (4)	
C8	0.25374 (8)	0.6941 (2)	0.31818 (10)	0.0218 (4)	
H8	0.2812	0.7597	0.2996	0.026*	
C9	0.26007 (8)	0.6719 (2)	0.39150 (10)	0.0251 (4)	
H9	0.2922	0.7226	0.4235	0.03*	
C10	0.21966 (8)	0.5759 (2)	0.41916 (10)	0.0236 (4)	
H10	0.2246	0.5635	0.4698	0.028*	
C11	0.17264 (8)	0.4985 (2)	0.37428 (9)	0.0195 (4)	
H11	0.1453	0.4334	0.3933	0.023*	
C12	0.16687 (7)	0.51933 (19)	0.30049 (9)	0.0164 (4)	
C13	0.12297 (7)	0.9420 (2)	0.35115 (9)	0.0193 (4)	
H13	0.1382	0.8312	0.361	0.023*	
C14	0.14981 (8)	1.0047 (2)	0.28869 (10)	0.0247 (4)	
H14A	0.136	1.1137	0.2776	0.037*	
H14B	0.1366	0.9377	0.2463	0.037*	
H14C	0.194	1.0031	0.3022	0.037*	
C15	0.14581 (8)	1.0381 (2)	0.41930 (10)	0.0247 (4)	
H15A	0.19	1.0448	0.428	0.037*	
H15B	0.1335	0.9862	0.4603	0.037*	
H15C	0.1286	1.145	0.4134	0.037*	
C16	0.01068 (8)	0.9163 (2)	0.40785 (9)	0.0213 (4)	
H16	0.0272	1.0094	0.4381	0.026*	
C17	0.03308 (9)	0.7674 (2)	0.45155 (11)	0.0316 (5)	
H17A	0.0223	0.7741	0.499	0.047*	
H17B	0.0771	0.7595	0.4573	0.047*	
H17C	0.0141	0.6734	0.4262	0.047*	
C18	−0.05797 (8)	0.9302 (2)	0.39410 (10)	0.0268 (4)	
H18A	−0.0761	0.8401	0.3649	0.04*	
H18B	−0.0705	1.0292	0.3684	0.04*	
H18C	−0.0714	0.9301	0.4401	0.04*	
C19	0.02934 (8)	0.7388 (2)	0.27707 (10)	0.0251 (4)	
H19A	0.0633	0.7196	0.2523	0.03*	
H19B	0.029	0.6525	0.3124	0.03*	
Cl1	−0.025597 (18)	1.29303 (5)	0.16367 (2)	0.01865 (11)	
N1	0.12485 (7)	0.45582 (17)	0.24411 (8)	0.0174 (3)	
Ni1	0	1.11320 (3)	0.25	0.01164 (9)	
P1	0.039792 (19)	0.93016 (5)	0.32401 (2)	0.01575 (11)	
H1N	0.0953 (9)	0.405 (2)	0.2501 (10)	0.019*	

### Atomic displacement parameters (Å^2^)

**Table:**

	*U*^11^	*U*^22^	*U*^33^	*U*^12^	*U*^13^	*U*^23^
C1	0.0171 (8)	0.0132 (8)	0.0203 (9)	0.0045 (7)	0.0080 (7)	0.0006 (7)
C2	0.0194 (9)	0.0193 (9)	0.0239 (10)	−0.0008 (7)	0.0046 (8)	−0.0053 (7)
C3	0.0245 (10)	0.0283 (10)	0.0183 (10)	0.0045 (8)	0.0045 (8)	−0.0027 (8)
C4	0.0257 (10)	0.0252 (9)	0.0214 (10)	0.0059 (8)	0.0114 (8)	0.0056 (8)
C5	0.0188 (9)	0.0181 (9)	0.0272 (10)	0.0014 (7)	0.0091 (8)	0.0037 (8)
C6	0.0143 (8)	0.0140 (8)	0.0214 (9)	0.0033 (7)	0.0060 (7)	0.0007 (7)
C7	0.0149 (8)	0.0147 (8)	0.0205 (9)	0.0027 (7)	0.0054 (7)	0.0006 (7)
C8	0.0184 (9)	0.0204 (9)	0.0274 (10)	−0.0012 (8)	0.0061 (8)	0.0000 (8)
C9	0.0198 (9)	0.0280 (10)	0.0248 (10)	0.0000 (8)	−0.0016 (8)	−0.0049 (8)
C10	0.0268 (10)	0.0265 (10)	0.0169 (9)	0.0076 (8)	0.0029 (8)	0.0026 (7)
C11	0.0221 (9)	0.0161 (9)	0.0222 (10)	0.0029 (7)	0.0089 (8)	0.0036 (7)
C12	0.0172 (8)	0.0127 (8)	0.0198 (9)	0.0039 (7)	0.0047 (7)	−0.0006 (7)
C13	0.0157 (8)	0.0182 (9)	0.0218 (9)	0.0040 (7)	−0.0020 (7)	0.0012 (7)
C14	0.0183 (9)	0.0281 (10)	0.0274 (10)	0.0031 (8)	0.0037 (8)	0.0027 (8)
C15	0.0173 (9)	0.0268 (10)	0.0270 (10)	−0.0005 (8)	−0.0027 (8)	−0.0019 (8)
C16	0.0212 (9)	0.0252 (10)	0.0165 (9)	−0.0046 (8)	0.0013 (7)	0.0039 (7)
C17	0.0337 (11)	0.0314 (11)	0.0278 (11)	−0.0026 (9)	0.0016 (9)	0.0124 (9)
C18	0.0220 (9)	0.0363 (11)	0.0219 (10)	−0.0054 (8)	0.0042 (8)	0.0041 (8)
C19	0.0311 (10)	0.0137 (8)	0.0276 (10)	0.0010 (8)	−0.0014 (8)	−0.0001 (8)
Cl1	0.0204 (2)	0.0144 (2)	0.0208 (2)	0.00193 (16)	0.00338 (17)	0.00393 (16)
N1	0.0162 (7)	0.0158 (7)	0.0218 (8)	−0.0033 (6)	0.0074 (6)	−0.0011 (6)
Ni1	0.01075 (15)	0.00869 (15)	0.01507 (16)	0	0.00161 (11)	0
P1	0.0167 (2)	0.0120 (2)	0.0169 (2)	−0.00081 (17)	−0.00073 (17)	0.00090 (17)

### Geometric parameters (Å, °)

**Table:**

C1—N1	1.386 (2)	C13—H13	1
C1—C2	1.397 (2)	C14—H14A	0.98
C1—C6	1.411 (2)	C14—H14B	0.98
C2—C3	1.383 (3)	C14—H14C	0.98
C2—H2	0.95	C15—H15A	0.98
C3—C4	1.398 (3)	C15—H15B	0.98
C3—H3	0.95	C15—H15C	0.98
C4—C5	1.376 (2)	C16—C18	1.524 (2)
C4—H4	0.95	C16—C17	1.534 (2)
C5—C6	1.399 (2)	C16—P1	1.8418 (18)
C5—H5	0.95	C16—H16	1
C6—C7	1.448 (2)	C17—H17A	0.98
C7—C8	1.394 (2)	C17—H17B	0.98
C7—C12	1.411 (2)	C17—H17C	0.98
C8—C9	1.382 (3)	C18—H18A	0.98
C8—H8	0.95	C18—H18B	0.98
C9—C10	1.399 (3)	C18—H18C	0.98
C9—H9	0.95	C19—C19^i^	1.505 (3)
C10—C11	1.384 (2)	C19—P1	1.8362 (18)
C10—H10	0.95	C19—H19A	0.99
C11—C12	1.390 (2)	C19—H19B	0.99
C11—H11	0.95	Ni1—Cl1	2.2221 (4)
C12—N1	1.387 (2)	N1—H1N	0.821 (19)
C13—C15	1.525 (2)	Ni1—P1^i^	2.1581 (5)
C13—C14	1.529 (3)	Ni1—P1	2.1581 (5)
C13—P1	1.8482 (17)	Ni1—Cl1^i^	2.2221 (4)
			
N1—C1—C2	129.44 (16)	H14B—C14—H14C	109.5
N1—C1—C6	108.91 (15)	C13—C15—H15A	109.5
C2—C1—C6	121.65 (16)	C13—C15—H15B	109.5
C3—C2—C1	117.26 (16)	H15A—C15—H15B	109.5
C3—C2—H2	121.4	C13—C15—H15C	109.5
C1—C2—H2	121.4	H15A—C15—H15C	109.5
C2—C3—C4	121.82 (17)	H15B—C15—H15C	109.5
C2—C3—H3	119.1	C18—C16—C17	111.69 (15)
C4—C3—H3	119.1	C18—C16—P1	111.96 (12)
C5—C4—C3	120.79 (17)	C17—C16—P1	112.49 (13)
C5—C4—H4	119.6	C18—C16—H16	106.8
C3—C4—H4	119.6	C17—C16—H16	106.8
C4—C5—C6	119.09 (17)	P1—C16—H16	106.8
C4—C5—H5	120.5	C16—C17—H17A	109.5
C6—C5—H5	120.5	C16—C17—H17B	109.5
C5—C6—C1	119.38 (16)	H17A—C17—H17B	109.5
C5—C6—C7	134.09 (16)	C16—C17—H17C	109.5
C1—C6—C7	106.53 (14)	H17A—C17—H17C	109.5
C8—C7—C12	119.53 (16)	H17B—C17—H17C	109.5
C8—C7—C6	133.67 (16)	C16—C18—H18A	109.5
C12—C7—C6	106.80 (14)	C16—C18—H18B	109.5
C9—C8—C7	118.98 (16)	H18A—C18—H18B	109.5
C9—C8—H8	120.5	C16—C18—H18C	109.5
C7—C8—H8	120.5	H18A—C18—H18C	109.5
C8—C9—C10	120.79 (17)	H18B—C18—H18C	109.5
C8—C9—H9	119.6	C19^i^—C19—P1	109.93 (8)
C10—C9—H9	119.6	C19^i^—C19—H19A	109.7
C11—C10—C9	121.37 (17)	P1—C19—H19A	109.7
C11—C10—H10	119.3	C19^i^—C19—H19B	109.7
C9—C10—H10	119.3	P1—C19—H19B	109.7
C10—C11—C12	117.70 (16)	H19A—C19—H19B	108.2
C10—C11—H11	121.1	C1—N1—C12	109.01 (14)
C12—C11—H11	121.1	C1—N1—H1N	126.9 (13)
N1—C12—C11	129.69 (15)	C12—N1—H1N	123.1 (13)
N1—C12—C7	108.71 (14)	P1^i^—Ni1—P1	88.61 (3)
C11—C12—C7	121.60 (16)	P1^i^—Ni1—Cl1^i^	168.757 (16)
C15—C13—C14	110.67 (15)	P1—Ni1—Cl1^i^	89.797 (16)
C15—C13—P1	114.66 (12)	P1^i^—Ni1—Cl1	89.797 (16)
C14—C13—P1	109.96 (12)	P1—Ni1—Cl1	168.758 (16)
C15—C13—H13	107.1	Cl1^i^—Ni1—Cl1	93.87 (2)
C14—C13—H13	107.1	C19—P1—C16	109.21 (9)
P1—C13—H13	107.1	C19—P1—C13	101.97 (8)
C13—C14—H14A	109.5	C16—P1—C13	106.35 (8)
C13—C14—H14B	109.5	C19—P1—Ni1	108.52 (6)
H14A—C14—H14B	109.5	C16—P1—Ni1	115.65 (6)
C13—C14—H14C	109.5	C13—P1—Ni1	114.22 (6)
H14A—C14—H14C	109.5		
			
N1—C1—C2—C3	−179.52 (17)	C6—C1—N1—C12	1.32 (18)
C6—C1—C2—C3	−0.4 (3)	C11—C12—N1—C1	177.09 (17)
C1—C2—C3—C4	−0.1 (3)	C7—C12—N1—C1	−2.04 (18)
C2—C3—C4—C5	0.2 (3)	C19^i^—C19—P1—C16	93.81 (18)
C3—C4—C5—C6	0.3 (3)	C19^i^—C19—P1—C13	−153.95 (17)
C4—C5—C6—C1	−0.7 (2)	C19^i^—C19—P1—Ni1	−33.06 (19)
C4—C5—C6—C7	179.56 (17)	C18—C16—P1—C19	−79.07 (14)
N1—C1—C6—C5	−179.89 (15)	C17—C16—P1—C19	47.67 (15)
C2—C1—C6—C5	0.8 (2)	C18—C16—P1—C13	171.60 (13)
N1—C1—C6—C7	−0.11 (18)	C17—C16—P1—C13	−61.66 (14)
C2—C1—C6—C7	−179.40 (15)	C18—C16—P1—Ni1	43.64 (14)
C5—C6—C7—C8	−0.7 (3)	C17—C16—P1—Ni1	170.38 (11)
C1—C6—C7—C8	179.53 (18)	C15—C13—P1—C19	−151.39 (13)
C5—C6—C7—C12	178.62 (18)	C14—C13—P1—C19	83.17 (14)
C1—C6—C7—C12	−1.10 (18)	C15—C13—P1—C16	−37.02 (15)
C12—C7—C8—C9	−1.3 (2)	C14—C13—P1—C16	−162.46 (12)
C6—C7—C8—C9	178.01 (17)	C15—C13—P1—Ni1	91.77 (13)
C7—C8—C9—C10	−0.2 (3)	C14—C13—P1—Ni1	−33.67 (14)
C8—C9—C10—C11	0.8 (3)	P1^i^—Ni1—P1—C19	10.07 (6)
C9—C10—C11—C12	0.0 (3)	Cl1^i^—Ni1—P1—C19	178.94 (7)
C10—C11—C12—N1	179.44 (16)	Cl1—Ni1—P1—C19	−71.87 (12)
C10—C11—C12—C7	−1.5 (2)	P1^i^—Ni1—P1—C16	−113.00 (7)
C8—C7—C12—N1	−178.60 (15)	Cl1^i^—Ni1—P1—C16	55.87 (6)
C6—C7—C12—N1	1.93 (18)	Cl1—Ni1—P1—C16	165.06 (10)
C8—C7—C12—C11	2.2 (2)	P1^i^—Ni1—P1—C13	123.06 (7)
C6—C7—C12—C11	−177.29 (15)	Cl1^i^—Ni1—P1—C13	−68.07 (6)
C2—C1—N1—C12	−179.46 (16)	Cl1—Ni1—P1—C13	41.12 (12)

Symmetry codes: (i) −*x*, *y*, −*z*+1/2.
